# Phosphatidylinositol- 3-kinase inhibitor induces chemosensitivity to a novel derivative of doxorubicin, AD198 chemotherapy in human bladder cancer cells *in vitro*

**DOI:** 10.1186/s12885-015-1930-5

**Published:** 2015-11-23

**Authors:** Dmitriy Smolensky, Kusum Rathore, Maria Cekanova

**Affiliations:** Department of Small Animal Clinical Sciences, College of Veterinary Medicine, The University of Tennessee, 2407 River Drive A122, Knoxville, TN 37996 USA; UT-ORNL Graduate School of Genome Science and Technology, The University of Tennessee, Knoxville, TN 37996 USA

**Keywords:** Doxorubicin, AD198, Bladder cancer, Apoptosis

## Abstract

**Background:**

Doxorubicin (Dox) is widely used to treat progressed bladder cancer after transurethral resection. The use of Dox-chemotherapy has been limited due to induced drug resistance and cumulative cardiotoxic effects. N-benzyladriamycin-14-valerate (AD198), a novel derivative of Dox, has a potential to become a more effective treatment than Dox by overcoming drug resistance and cardio-toxicity as shown in the rodent model of lymphoma *in vivo*. The purpose of this study was to compare the efficacy of Dox and AD198 and explore their mechanisms in inhibition on human bladder cancer cells *in vitro*.

**Methods:**

We evaluated the effects of Dox and AD198 on cell viability of human transitional cell carcinoma (TCC) cell lines T24 and UMUC3 by MTS assay *in vitro*. The effects of Dox and AD198 on cell apoptosis were determined by caspase 3/7 assay, generation of reactive oxygen species (ROS), and Western Blotting (WB) analysis.

**Results:**

AD198 was more effective than Dox in inhibition of cell viability of T24 and UMUC3 cells *in vitro*. Both Dox and AD198 significantly increased the generation of ROS and induced apoptosis in caspase-dependent and -independent manner in T24 and UMUC3 cells. AD 198 induced significantly higher production of ROS as compared to Dox in human TCC cells. Dox and AD198 activated the pro-apoptotic p38 MAPK pathway; however, on the other hand also increased phosphorylation of AKT, an anti-apoptotic signaling pathway, in T24 and UMUC3 cells. Combined treatment of PI3K inhibitor (LY294002) with Dox or AD198 inhibited cell viability of T24 and UMUC3 cells more effectively than any of drug treatments alone.

**Conclusions:**

These data suggest that AD198 as novel derivative of Dox, could be a used as effective treatment for bladder cancer. Dox and AD198 induced PI3K/AKT signaling pathway that is a one of the indicators of pro-survival and possible drug-resistance mechanisms of chemotherapies in bladder cancer. Combined therapies of Dox or AD198 with inhibitors of PI3K/AKT signaling pathway might lead to more effective treatment outcome for patients diagnosed with bladder cancer based on our *in vitro* experiments.

## Background

Bladder cancer is the 6th most common cancer in the United States, with high rates of recurrence [1, 2]. While the exact reasons are unknown, bladder cancer presents itself four times more in males than females [1, 3]. Urothelial cancer, also known as transitional cell carcinoma (TCC), is the most common type of bladder cancer in the western world and accounts for over 90 % of all bladder cancer cases [2, 4]. The 5-year survival rates for patients diagnosed with the earlier stages of the bladder cancer are 69.2 %. However; the 5-year survival rates for patients diagnosed with invasive bladder cancer at stage IV are only 5.5 % [2]. The biggest challenges in treatment of bladder cancer are the high rates of reoccurrence and progression from non-invasive to invasive stages of bladder cancer. The invasion of bladder cancer into the muscle layer of the bladder serves as major prognostic marker for the development of the treatment plan [3]. Tobacco products have been determined to be the highest environmental risk factor for developing bladder cancer [2]. Other environmental risk factors for bladder cancer include occupational exposure and chemotherapy drugs, while non-environmental risk factors include age, gender, race, obesity and family history [1, 2, 5].

Superficial bladder cancer is well managed by transurethral resection (TUR), followed by an intravenous or intravesical (directly into the bladder) administrations of chemotherapeutic treatment, such as mitomycin, epirubicin or doxorubicin (Dox) [6–8]. The intravesical injection of bacillus Calmette-Guérin (BCG), as adjuvant immunotherapy, activates immune system in the patient and greatly increases progression free survival rates [8, 9]. The management treatment for patients with muscle invasive bladder cancer is usually a radical cystectomy (removal of whole bladder) mostly followed by adjuvant therapy, such as chemotherapy and radiation therapy [3]. Chemotherapy protocols without radiation include: cisplatin alone, or cisplatin with 5-flourouracil or mitomycin with 5-flourouracil [10]. Chemotherapy protocols in conjunction with radiation include: gemcitabine with cisplatin, the MVAC protocol - methotrexate, vinblastine, Dox (Adriamycin), cisplatin or combination of carboplatin with either paclitaxel or docetaxel [10].

Dox is an anthracycline antibiotics and is one of the most widely used anti-cancer drugs [8, 11]. Dox interacts with topoisomerase II (TOPOII) [12–14] and induces apoptosis through production of reactive oxygen species (ROS) and by inducing DNA damage in bladder cells [15]. Dox induces ROS production through p53-dependent and p53-independent mechanisms [16, 17]. However, other mechanisms of Dox action remain unclear. While Dox has been successful in treating patients diagnosed with different cancers, long term use of Dox has two major setbacks. Firstly, Dox induces drug resistance through the upregulation of the multi-drug resistance 1 (*MDR1*) gene, also known as p-glycoprotein in the cell [18, 19]. Secondly, long term use of Dox has been linked to acute cardiotoxicity [20].

N-benzyladriamycin-14-valerate (AD198), one of the derivatives of Dox, which shows improvement in cardiotoxicity as compared to Dox [21]. The addition of an N-benzyl ring improves the lipophilic properties of AD198 and allowing rapid localization of AD198 in the cytoplasm of cells [21]. The structural similarity of a moiety of the AD198 molecule to diacylglycerol (DAG) allows AD198 to interact with the regulatory subunit of PKC-δ by releasing the catalytic subunit [22]. The released PKC-δ catalytic subunit attributes to its cytotoxic effects by inducing mitochondrial membrane depolarization and inducing ROS production [22]. In cardiomyocytes, AD198 activates PKC-ε, which attributes to cardio-protective effects by Dox-induced ROS production [23]. AD198 has been shown to be effective in inhibition of cell growth of mouse lymphoma and multiple-myeloma models [24]. In addition, AD198 is more effective in inhibition of cell proliferation and inducing apoptosis in canine TCC and osteosarcoma primary cell lines than Dox through the p38 MAPK signaling pathway [25]. Cardio-toxicity, which is a major concern for patients receiving Dox treatment, has not been detected when rats were treated with AD198 [26]. In rats, low dose administration of AD198 after Dox-induced cardiotoxicity, attenuated markers of cardiotoxicity, when compared to Dox alone [27]. This cardio-protective property of AD198 has been attributed to activation of PKC-ε, while PKC-ε knockout mice did not benefit from cardio protective effects of AD198 [23].

AD198 has not been evaluated for its potential use in bladder cancer treatment. So in this study, we evaluated the efficacy and mechanisms of Dox and its derivative, AD198 on cell proliferation and apoptosis in human UMUC3 and T24 TCC cell lines *in vitro*.

## Methods

### Reagents and antibodies

Unless otherwise stated, all reagents and media were purchased from Fisher Scientific (Pittsburgh, PA.) Dox and LY294002 were purchased from Sigma-Aldrich, St. Louis, MO. N-benzyladriamycin-14-valerate (AD198) was a kind gift from Dr. Leonard Lothstein (The University of Tennessee, Health Science Center in Memphis) [21]. The following antibodies were purchased from Santa Cruz Biotechnology, (Santa Cruz, CA): Actin-HRP, p-ERK1/2, ERK1/2, AKT and p38. The following antibodies were purchased from Cell Signaling (Boston, MA): PARP, p-AKT (Serine 473 and Threonine 308), p-GSK3β and p-p38.

### Cell lines

Human transitional cell carcinoma T24 and UMUC3 cell lines were purchased from ATCC (Manassas, VA). The cells were grown in the following media: T24 in McCoy’s media, UMUC3 in MEM media containing 10 % FBS and penicillin/streptomycin mixture (Fisher Scientific, Pittsburgh, PA) in 37 °C and 5 % CO_2_.

### Proliferation assay

Cells were plated in 96-well plates at 5 × 10^3^ cells per well and allowed to attach for 24 h. After seeding, cells were treated with AD198 or Dox in dose-dependent manner in complete media for additional 48 h. DMSO was used as control. For co-treatment with PI3K inhibitor (LY294002), the cells were pretreated with 20 μM LY294002 for 30 min prior to AD189 or Dox treatments followed by combined treatment for 48 h. After treatment, the viability of cells was measured using CellTiter 96® Aqueous One Solution Cell Proliferation Assay (Promega, Madison, WI) according to manufacturer’s protocol. Briefly, 20 μL of the MTS reagent was added to each well and allowed to incubate at 37°C for 1 h. Absorbance was measured at 490 nM using a plate reader (Bio-Tek instruments, Winooski, VT). The treatment results were normalized to the DMSO controls.

### Reactive Oxygen Species (ROS) assay by flow cytometry

After cells were treated with Dox or AD for 24 h, the cells were incubated with 5 μM dihydrogen-dichlorodihydro-fluorescein-diacetate (H_2_DCF-DA; Life Technologies, Grand Island, NY) for 1 h. The cells were then washed twice with PBS and trypsinized. The trypsin was neutralized and the collected cells were centrifuged at 5000 rpm for 5 min. The cell pellet was re-suspended in 1 mL of PBS and the fluorescence was measured at 485 nM excitation and 530 nM emission using flow cytometer (BD Accuri® BD Sciences, San Jose, CA). The treatment results were normalized to the DMSO controls.

### Caspase-3/7 assay

Cells were plated in 6-well plates at 5 × 10^5^ cells per well. After 24 h, cells were treated with AD198 or Dox for additional 24 h. After treatment, cells were washed twice with PBS, and cell lysates were harvested using RIPA buffer. Protein concentration was measured using Bradford BCA assay. Forty micrograms of proteins were used for detection of caspases 3/7 following the Caspase Glo® 3/7 Substrate protocol (Promega). After 1 h incubation with reagents, the luminescence was measured using FLx800 plate reader (Bio-Tek instruments, Winooski, VT). The treatment data were normalized to the DMSO controls.

### Western blot

Cells were plated at 1.5 × 10^6^ cells per 10-cm plate. Twenty four hours after plating, the cells were treated with AD or Dox for 24 h in dose-depend manner (0.1, 0.5 and 1.0 μM). For co-treatment with PI3K inhibitor (LY294002), the cells were pretreated with 20 μM LY294002 for 30 min prior to AD or Dox co-treatments for additional 24 h. After treatment, the cells were washed twice with PBS and lysed using cold RIPA buffer containing protease/phosphatase inhibitors. The cell lysates were kept at −80 °C until further analysis. Protein concentrations were measured using the BCA protein assay. Equal amount of proteins (60 μg) were loaded onto SDS-PAGE gels and transferred to a nitrocellulose membrane. After blocking, the membranes were incubated with primary antibodies at 4 °C overnight and then incubated with horseradish peroxidase-conjugated secondary antibodies for 1 h at room temperature. The immunoreactive bands were visualized using enhanced chemiluminescence system (Fisher) and acquired using the ImageQuant LAS4000 (GE Life Sciences, Pittsburgh, PA.) The densitometry analysis of proteins were normalized to actin from three independent WB experiments using ImageJ (NIH) software.

### Statistical analysis

Statistical analysis were conducted using paired Student *t-*test to established significance. Results were considered statistically significant at *p ≤ 0.05, **p ≤ 0.01, ***p ≤ 0.001 when treatments were compared to control groups and ^#^*p* ≤ 0.05, ^##^*p* ≤ 0.01, ^###^*p* ≤ 0.001 when Dox groups were compared to AD198 groups at the same doses, or when Dox or AD198 groups were compared to LY + Dox or LY + AD198 groups.

## Results

### DOX and AD198 inhibited cell viability of human TCC cells

**Fig. 1:**
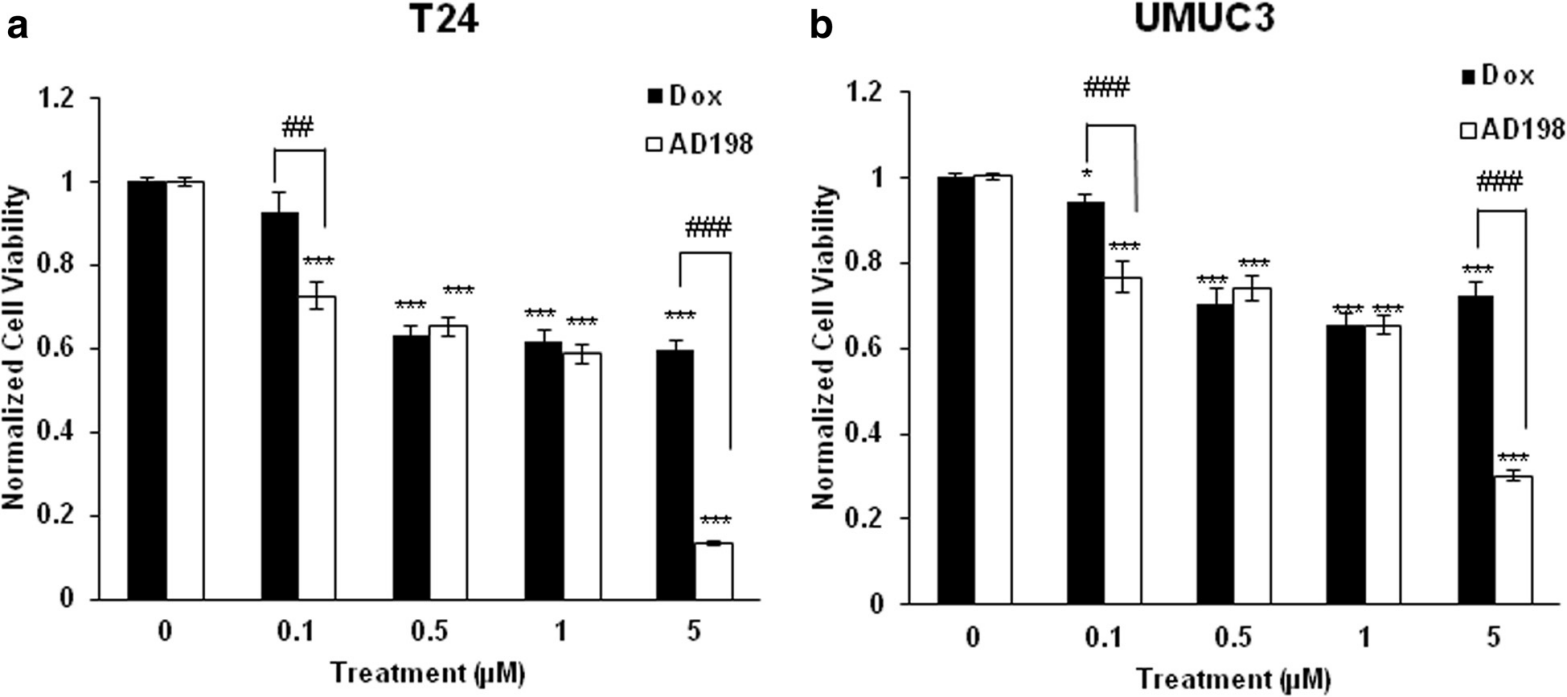
DOX and AD198 inhibited cell viability of human TCC cells. (**a**) Human urinary bladder transitional cell carcinoma (TCC) cells T24 and (**b**) UMUC3 cells were treated with Dox (black bars) and AD198 (white bars) at 0.1, 0.5, 1 and 5 μM for 48 h and compared to control groups. Cell proliferation was determined by MTS assay and relative cell growth rate was normalized to control counterpart. Values represent mean ± S.E. of four replicates from three independent experiments. Paired Student *t*-tests were used to compare Dox and AD198 treatment to control; * *p* ≤ 0.05 and *** *p* ≤ 0.001. Paired Student *t*-tests were used to compare among Dox and AD198 group at the same dose treatment; ^##^ *p* ≤ 0.01 and ^###^ *p* ≤ 0.001

Human TCC cell lines, T24 and UMUC3 were treated with 0.1, 0.5, 1 and 5 μM of Dox and AD198 for 48 h, as shown in Fig. 1. Both, Dox and AD198, significantly reduced the proliferation of T24 (Fig. 1a) and UMUC3 (Fig. 1b) cells in dose-dependent manner. AD198 was significantly more effective in inhibition of cell viability in both T24 and UMUC3 cells as compared to Dox at the concentrations of 0.1 and 5 μM.

### Dox and AD198 induced ROS production in human TCC cells

**Fig. 2:**
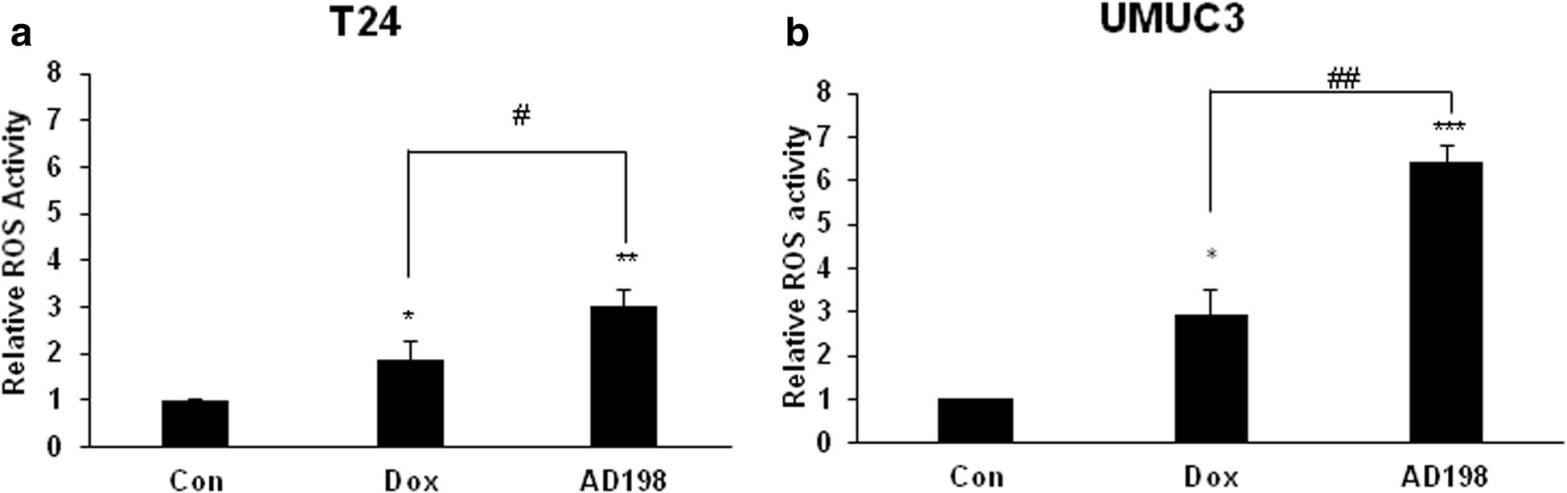
Dox and AD198 induced ROS in human TCC cells. (**a**) T24 and (**b**) UMUC3 cells were treated with 1μM Dox and AD198 for 24 h and ROS levels were measured with dihydrogen-dichlorodihydro-fluoresceindiacetate assay; percent of ROS positive cells were measured and normalized to the control. Values represent mean ± S.E. of three independent experiments. Paired Student t- tests were used to compare Doxand AD198 treatments to controls, *p ≤ 0.05, **p ≤ 0.01, ***p ≤ 0.001. Paired Student t-tests were used to compare among Dox and AD198 group at the same dose treatment; #*p* ≤ 0.05 and ##*p* ≤ 0.01

The effects of Dox and AD198 on generation of cellular ROS was evaluated using H_2_DCF-DA assay. Dox and AD198 both significantly increased ROS in T24 (Fig. 2a) and UMUC3 (Fig. 2b) cells after 24 h treatment; and in addition, AD198 showed significantly higher activation of ROS production as compared to DOX with 3-fold vs 2-fold increase in T24 cells and 6-fold vs 3-fold increase in UMUC3; respectively (*p ≤ 0.05 in T24 and **p ≤ 0.01 in UMUC3) as shown in Fig. 2.

### Dox and AD198 induced apoptosis in human TCC cells through activation of caspase cascade

**Fig. 3:**
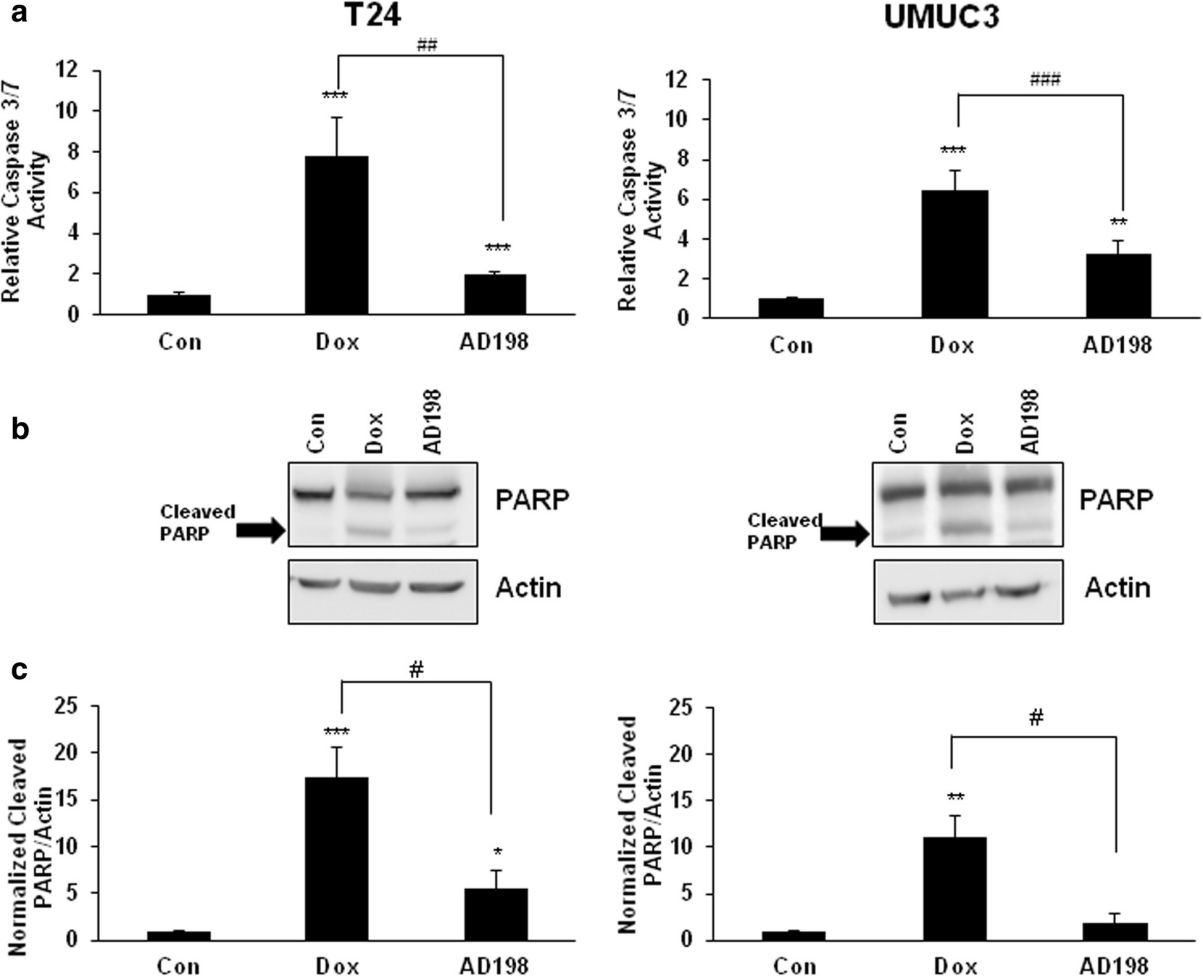
Dox and AD198 induced apoptosis in human TCC cells. **a** T24 and UMUC3 cells were treated with 1 μM Dox and AD198 for 24 h and caspase-3/7 activities were measured using the Caspase-Glo 3/7 luminescence assay. Relative caspase-3/7 activities were normalized to control. Values represent mean ± SE of three independent experiments. Paired Student *t-*test were used to compare treatment to control **p ≤ 0.01, ***p ≤ 0.001. Student *t-*tests were used to compare among Dox and AD198 treatments ^##^ *p* ≤ 0.01, ^###^ *p* ≤ 0.001. **b** T24 and UMUC3 cells were treated with 1 μM Dox and AD198 for 24 h. The expression of PARP (cleaved fragment) was evaluated by WB analysis. Actin was used as a loading control. **c** Densitometry evaluation of cleaved PARP protein bands from WB analysis was done using ImageJ software. Values represent mean ± S.E. of measured densitometry of each band from three independent experiments. Paired Student *t-*tests were used to compare controls to Dox and AD198 treatments, *p ≤ 0.05, **p ≤ 0.01, and ***p ≤ 0.001. Paired Student *t*- tests were used to compare Dox to AD198 treatment, ^#^ *p* ≤ 0.05

The effects of Dox and AD198 on cell apoptosis were evaluated using the caspase-3/7 activities assay. Dox and AD198 both increased apoptosis in T24 and UMUC3 cells; however, Dox showed significantly higher caspase activation than AD198 in both TCC cell lines (^##^*p* ≤ 0.01 in T24 and ^###^*p* ≤ 0.001 in UMUC3) as shown in Fig. 3a.

Poly (ADP-ribose) polymerase (PARP) is a family of proteins involved in genomic stability and are downstream targets, which are cleaved by caspases to produce 89 and 24 kD fragments [28, 29]. The presence of degraded PARP is generally considered as a marker of apoptosis [28]. Dox and AD198 (1 μM) treatments increased a cleavage of PARP in both tested cells as confirmed by WB analysis (Fig. 3b). Densitometry values of cleaved PARP protein after Dox and AD198 treatments were normalized to actin and then to control group as shown in Fig. 3c. A statistically significant increases in PARP cleavage (***p ≤ 0.001 and **p ≤ 0.01) by 17- and 11-fold were observed with Dox treatment as compared to control treatments in T24 and UMUC3 cells; respectively (Fig. 3c). Also Dox significantly increased PARP cleavage by 3- and 4-fold (^#^*p* ≤ 0.05) as compared to AD198 treatment in T24 and UMUC3 cells; respectively (Fig. 3c).

### Dox and AD198 activated PI3K/AKT signaling pathway in human TCC cells

**Fig. 4:**
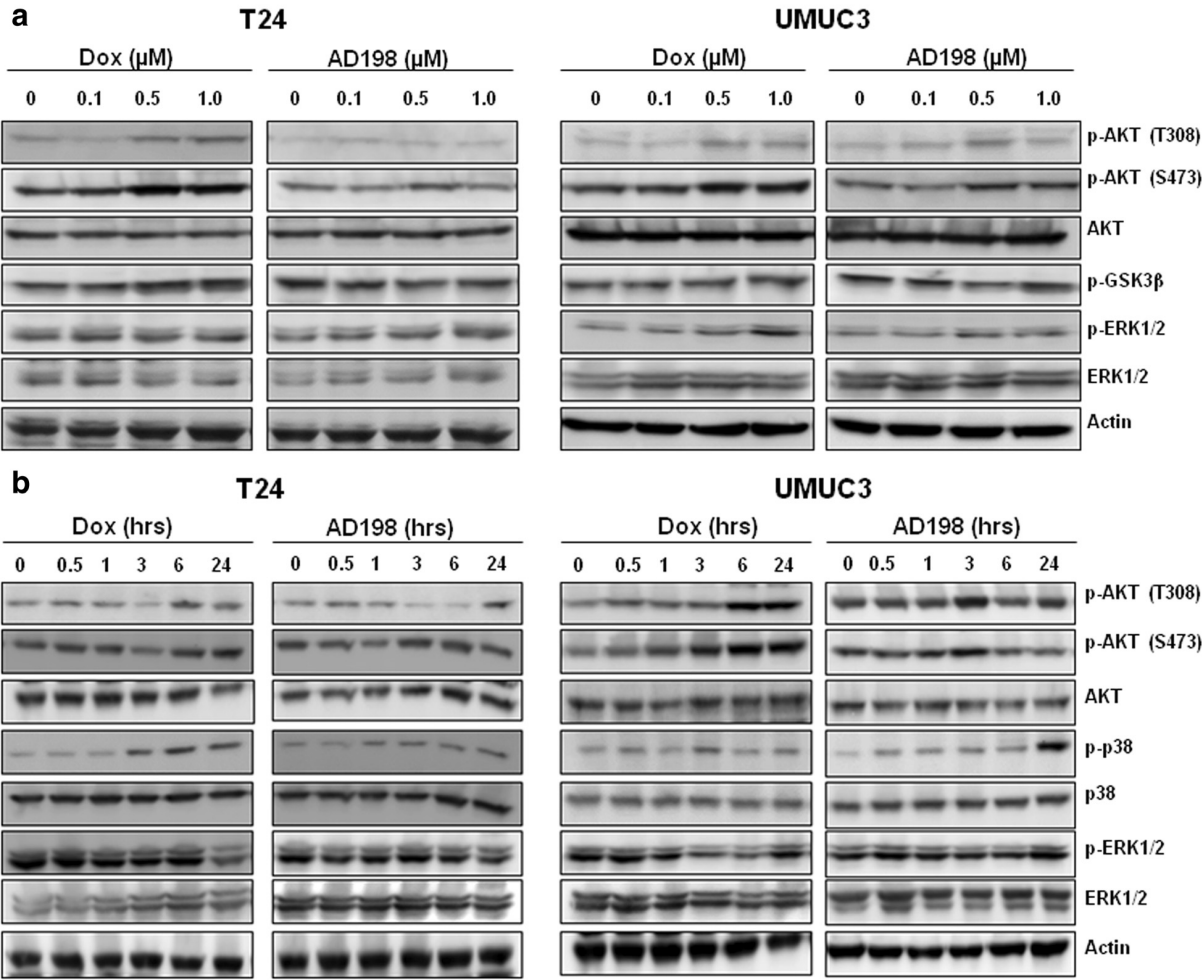
Dox activated AKT signaling pathway in human TCC cells in dose- and time-dependent manner. **a** T24 and UMUC3 cells were treated with 0.1, 0.5 and 1 μM Dox and AD198 for 24 h. Protein levels of p-AKT (T308), p-AKT (S473), AKT, p-GSK-3β, p-ERK1/2 and ERK1/2 were detected by WB. Actin was used as a loading control. **b** T24 and UMUC3 cells were treated with 1 μM Dox and AD198 for 0, 0.5, 1, 3, 6 and 24 h. Protein levels of p-AKT (T308), p-AKT (S473), AKT, p-p38, p38, p-ERK1/2 and ERK1/2 were detected by WB. Actin was used as a loading control

To better understand the mechanisms of AD198 and Dox-induced cell growth inhibition in T24 and UMUC3 cells, we investigated role of PI3K/AKT and MAPK signaling pathways. Dox increased the phosphorylation of AKT protein at both Ser473 and Thr308 sites in T24 and UMUC3 cells in time- and dose-dependent manner (Fig. 4). GSK-3β is a critical downstream element of the PI3K/AKT cell survival pathway, and when phosphorylated, its pro-apoptotic function is attenuated by AKT [30]. Dox increased phosphorylation of GSK-3β in dose-dependent manner as shown in Fig. 4a. There was no significant increase in the phosphorylation of ERK1/2 in either TCC cells when treated with Dox or AD198 (Fig. 4a). The p38 MAPK has been shown to be activated by ROS and plays a vital role in apoptosis [31, 32]. Both Dox and AD198 increased phosphorylation of p38 MAPK in a time-dependent manner with increased activation at 1–3 h after AD198 and Dox treatments in T24 cells (Fig. 4b). In UMUC3 cells, the activation of p38 was seen to be higher with AD198 than with Dox treatments, confirming previous results of higher ROS production by AD198 (Fig. 4b).

### Inhibition of PI3K/AKT signaling pathway sensitizing the cytotoxic effects of Dox and AD198 in human TCC cells

**Fig. 5:**
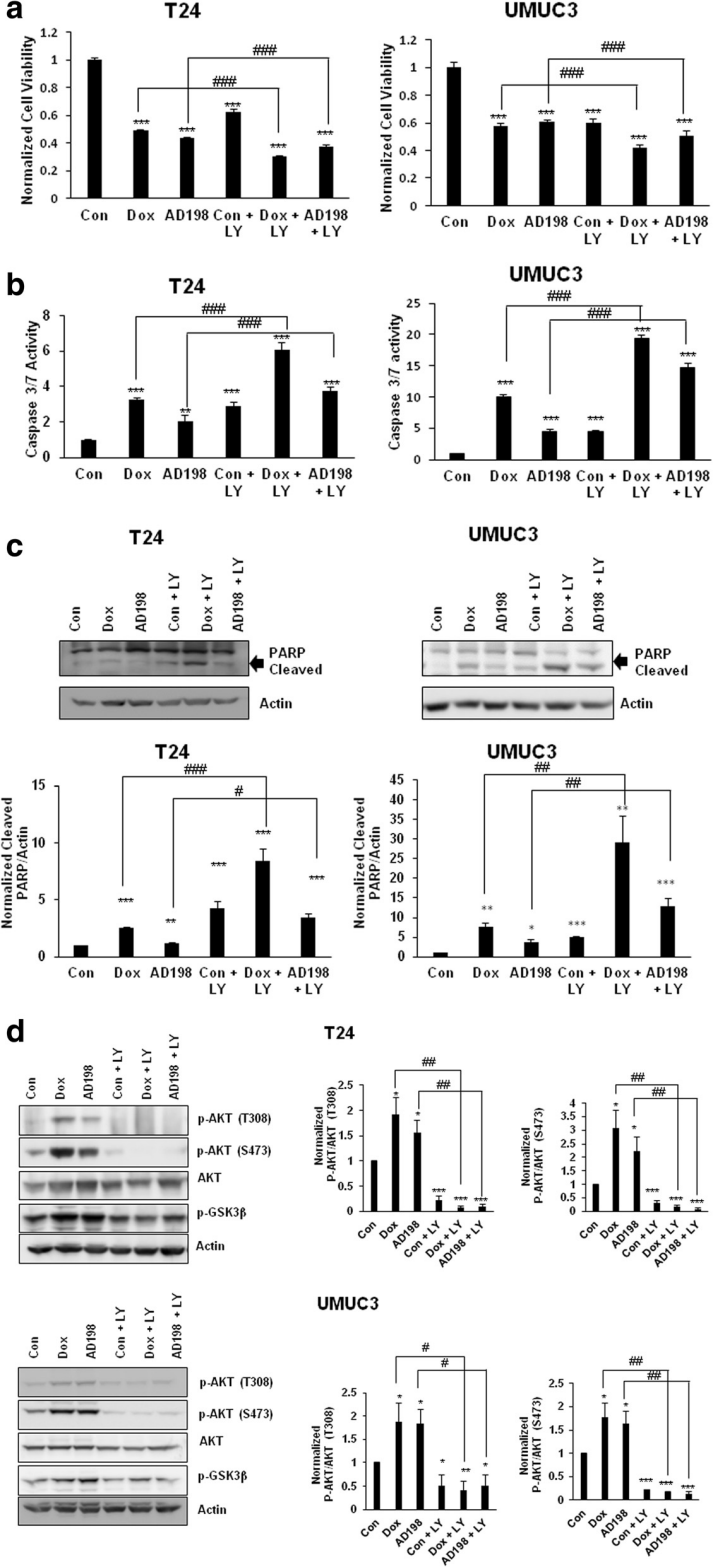
Inhibition of AKT1 signaling pathway sensitizing the cytotoxic effects of Dox and AD198 in human TCC cells. **a** T24 and UMUC3 cells were treated with Dox and AD198 (1 μM) with and without LY294002 (LY, 20 μM) for 48 h and compared to control groups. Cell proliferation was determined by MTS assay and relative cell growth rate was normalized to control counterpart. Values represent mean ± SE of four replicates from three independent experiments. Paired Student *t-*tests were used to compare DOX and AD198 treatments to control; ***p ≤ 0.001. Paired Student *t-*tests were used to compare Dox to Dox + Ly and AD198 to AD198 + LY treatment, ^###^ *p* ≤ 0.001. **b** T24 and UMUC3 cells were treated with DOX and AD198 (1 μM) with and without LY294002 (20 μM) for 24 h and caspase activities were measured using the Caspase-Glo 3/7 luminescence assay. Relative caspase activities were normalized to control. Values represent mean ± SE of three independent experiments. Paired Student *t-*tests were used to compare treatment to control **p ≤ 0.01, ***p ≤ 0.001. Paired Student *t-*tests were used to compare Dox to Dox + Ly and AD198 to AD198 + LY treatments, ^###^ *p* ≤ 0.001. **c** T24 and UMUC3 cells were treated with Dox and AD198 (1 μM) with and without LY294002 (20 μM) for 24 h. The expression of PARP (cleaved fragment) were evaluated by WB analysis. Actin was used as a loading control. Densitometry evaluation of PARP protein bands from WB analysis was done using ImageJ software. Values represent mean ± S.E. of measured densitometry of each protein’s band from three independent experiments. Paired Student *t-*tests were used to compare controls to Dox and AD198 treatments, *p ≤ 0.05, **p ≤ 0.01, and ***p ≤ 0.001. Paired Student *t-*tests were used to compare Dox to AD198 treatment, ^#^ *p* ≤ 0.05, ^##^ *p* ≤ 0.05, and ^###^ *p* ≤ 0.001 **d** T24 and UMUC3 cells were treated with Dox and AD198 (1 μM) with and without LY294002 (20 μM) for 24 h. The expression of p-AKT (T308), p-AKT (S473), AKT1 and p-GSK-3β proteins were evaluated by WB analysis. Actin was used as a loading control. Densitometry evaluation of p-AKT (T308), p-AKT (S473) protein bands from WB analysis was done using ImageJ software. Values represent mean ± S.E. of measured densitometry of each band from three independent experiments. Paired Student *t-*tests were used to compare controls to Dox and AD198 treatments, *p ≤ 0.05, **p ≤ 0.01, and ***p ≤ 0.001. Paired Student *t-*tests were used to compare Dox to Dox + LY or AD198 to AD198 + LY treatments, ^#^ *p* ≤ 0.05, ^##^ *p* ≤ 0.01

Dox and AD198 activated AKT pro-survival signaling pathway that is an indicator of resistance of cells to chemotherapy. To confirm our hypothesis, we tested the effects of PI3K inhibitor, LY294002, in combination with Dox or AD198 on cell growth of TCC cells. Co-treatment with LY294002 increased the anti-proliferative effects of both Dox and AD198 in T24 and UMUC3 cells (Fig. 5a). In order to further investigate the PI3K/AKT inhibitor’s chemosensitizing effects to Dox and AD198 chemotherapies, we measured caspase-3/7 activities and PARP cleavage. Indeed, co-treatment of Dox and AD198 with PI3K inhibitor, LY294002, increased caspase-3/7 activation and PARP cleavage in both T24 and UMUC3 cells as shown in Fig. 5b and c. LY294002 inhibited the AD198- and Dox-induced phosphorylation of AKT at Thr308 and Ser473 sites as shown in Fig. 5d. In addition, higher levels of active (unphosphorylated) GSK3β were present when T24 and UMUC3 cells were co-treated with Dox or AD198 and LY294002.

## Discussion

Dox has been used to treat human bladder cancer for over three decades and continues to be one of the most common chemotherapeutic agent [33]. Dox is not as effective alone as it is in combination with other drugs; however, Dox increases side effects and decreases completion of regimen due to intolerances by the patients [34]. Another setback of Dox in the treatment of bladder cancer is the development of drug resistance by up-regulation of p-glycoprotein efflux transporter protein expressions. The established Dox-resistant bladder cancer cell lines, KK47/ADM, shows that complete reversal of resistance was not possible even when Dox was used in combination with a sensitizing agent, verapamil [35]. The development of novel derivatives of Dox may overcome those Dox adverse events, and even exceed its anti-cancer effects [36].

A novel derivative of Dox, AD198 has been developed by Dr. Lothstein’s group [21]. AD198 is a highly lipophilic drug, which rapidly localizes to the cellular cytoplasm and it has been shown to circumvent efflux transport by p-glycoprotein in Dox-resistant macrophage cells [21, 37]. AD198 has been shown to overcome Bcr-Abl pro-survival signaling pathway in human leukemia cells through the activation of ERK1/2 and STAT-5 followed by cytochrome C release and apoptosis [38]. Breast and ovarian cancer cell lines, which are resistant to Dox due to p-glycoprotein expression, have been shown to rapidly accumulate AD198 in the cytoplasm. The efficacy of AD198 to inhibit cell growth is comparable to Dox treatment in non-resistant cells [39]. Our results are in agreement with this study and confirmed that AD198 anti-proliferative effect was similar and compared to Dox in T24 and UMUC3 cells. This might be relevant for the intravesicular treatment of bladder cancer, where a high dose of the Dox is used (~200 mM) [8].

It has been shown that AD198 and Dox have a similar effect in generating ROS in murine cardiomyocytes [23]. However; ROS production was induced by AD198 more than by Dox in both tested human bladder cell lines (Fig. 2). In addition to ROS production in the cytoplasm, Dox induces DNA damage via Topoisomerase II, while AD198 mainly functions in the cytoplasm by increasing ROS and activating PKC-δ [40]. In mouse myeloid cells, AD198 induces apoptosis through activation of PKC-δ and is not effected by the expression of Bcl-2 [22]. AD198 acts through PKC-δ-independent manner in TRAF-3 deficient mouse B-lymphoma cells through the suppression of oncogenic protein c-Myc [24]. AD198 might be beneficial for treatment of c-Myc overexpressing cancer cells. While AD198 had an equal or greater anti-proliferative and ROS generating effects than Dox in TCC, it showed significantly less caspase activation and PARP cleavage in both T24 and UMUC3 (Fig. 3a–c). It has been previously shown that AD198 induces cytochrome-C release and initiate mitochondrial-activated apoptosis, even when caspase activation is blocked by a pan-caspase inhibitor, Z-VAD-FMK [22]. In contrast, Dox has been shown to function in a caspase-dependent manner in T-leukemia cells and apoptosis was inhibited when Jurkat cells were treated with Z-VAD-FMK [41]. Dox has been shown to induce apoptosis in ROS-independent manner in cardiomyocytes [42]. Our data provides evidence that AD198 induced apoptosis in caspase-dependent and -independent pathways. Dox, on the other hand, induce apoptosis primarily through caspase-dependent pathway.

To further investigate the differences in mechanisms between Dox and AD198 action in TCC cells, we investigated PI3K/AKT and MAPK signaling pathways. AKT has been shown to increase drug resistance in other cancers and PI3K itself can contribute to expression of multidrug resistance protein 1 (MDR1) to induce drug resistance [30, 43, 44]. Dox activates the PI3K/AKT pathway in several cancers including ovarian, hepatic and breast cancer cells [45–47]. Activation of AKT by Dox has been linked to the presence of human epidermal growth factor receptor 3 (HER3, ERBB3 ) in ovarian cancer and was attenuated by the addition of tyrosine kinase inhibitors lapatinib and/or erlotinib [45]. In our study, Dox more efficiently phosphorylated AKT (Ser473 and Thr308) and its downstream target GSK3β than AD198 in dose- and time- dependent manner (Fig. 4a and b). PI3K/AKT pathway has been greatly implicated in the progression and prognosis in bladder cancer patients [48]. The growth factor receptors including ERBB2, ERBB3 and EGFR have been found to be altered or amplified in bladder cancer and have the potential to activate PI3K/AKT signaling pathway [49]. PI3K mutation is inversely associated with later stages, indicating that mutation of PI3K is not crucial to bladder cancer progression [50, 51]. AKT after activation by PIP3, has a wide range of downstream targets including activation of anti-apoptotic targets such MDM2 and mTOR as well as deactivation of apoptotic targets such as BAD, GSK3β and TSC1 [52, 53]. It has been shown that inhibition of PI3K sensitizes TCC cells to Dox chemotherapy and lowers the IC50 of Dox when used in combination with LY294002 [54]. We confirmed that co-treatment of PI3K/AKT inhibitor with Dox or AD198 reduced cell proliferation more efficiently than Dox or AD198 treatments alone in tested human bladder cells (Fig. 5a). Furthermore, co-treatment of LY294002 with either Dox or AD198 induced an activation of caspase-3/7 activity and cleavage of PARP than AD198 or Dox treatments alone (Fig. 5b and c). In T24 and UMUC3 cells, phosphorylation of GSK3β was decreased by co-treatments, indicating that its pro-apoptotic function was restored (Fig. 5d), while either Dox or AD198 alone increase phosphorylation of GSK3β indicating an anti-apoptotic resistance activated by AKT signaling. The tumor suppressor PTEN (phosphatase and tensin homolog deleted on chromosome 10) antagonizes the PI3K/AKT signaling pathway and mutation or decrease of *PTEN* expression has been shown to be as a poor prognostic marker in breast cancer patients [55]. The *Pten* gene therapy in mice increases tumor sensitivity to Dox therapy *in vivo* [56]. T24 cells have mutation in *PTEN* gene, but cells express PTEN protein, while intragenic deletion of *PTEN* gene in exons1-8 in UMUC3 cells results in no PTEN protein expression [57]. We have observed that phosphorylation of AKT1 levels were much higher in UMUC3 cells than T24 probably due to absence of PTEN (data not shown).

## Conclusions

In conclusion, we have shown that AD198 has comparable anti-proliferative efficacy as Dox in tested human TCC cell lines *in vitro*. AD198 was more effective in induction of ROS production. AD198 induced apoptosis in caspase-dependent and -independent pathways. Dox, on the other hand, induced apoptosis primarily through caspase-dependent pathway. Both drugs activated PI3K/AKT signaling pathway, which may explain a common mechanisms of bladder cancer to acquire a drug resistance. The inhibition of the PI3K/AKT pathway plays an important role in increasing the effectiveness of Dox and AD198 treatments in human bladder cancer cells in vitro. AD198 a novel derivative of Dox, with no cardio-toxic effects as has been shown in mice *in vivo* model, may be a new candidate for the replacement of Dox treatment in bladder cancer. Further investigations using rodent animal model of bladder cancer *in vivo* are required to support these *in vitro* findings.

## Abbreviations

AD198: N-benzyladriamycin-14-valerate
AKT: V-akt murine thymoma viral oncogene homolog 1
BCG: bacillus calmette-guerin
Dox: doxorubicin
ERK: extracellular signal regulated kinases
GSK3β: glycogen synthase kinase 3 beta
H2DCF-DA: dihydrogen-dichlorodihydro-fluorescein-diacetate
LY: LY294002
MVAC: methotrexate, vinblastine, Dox and cisplatin chemotherapy protocol
PARP: poly (ADP-ribose) polymerase
PTEN: phosphatase and tensin homolog deleted on chromosome 10
ROS: reactive oxygen species
TCC: transitional cell carcinoma
TOPOII: topoisomerase II
TUR: trans-urothelial resection
ERBB: human epidermal growth factor receptor
MDR1: multidrug resistance protein 1
PI3K: phosphatidylinositol-3-kinase
